# Two Cases of Menkes Disease With Similar Intracranial Arterial Tortuosity on Brain Magnetic Resonance Imaging

**DOI:** 10.7759/cureus.74280

**Published:** 2024-11-22

**Authors:** Shichiro Katase, Kazuhiro Tsuchiya, Nanako Habu, Ichiro Hada, Shohei Takahashi, Ryota Kurayama, Miho Gomyo, Masanaka Watanabe, Kaori Iwamoto, Kenichi Yokoyama

**Affiliations:** 1 Department of Radiology, Kyorin University, Faculty of Medicine, Tokyo, JPN; 2 Department of Radiology, Kyorin University Suginami Hospital, Tokyo, JPN; 3 Department of Pediatrics, Kyorin University Suginami Hospital, Tokyo, JPN

**Keywords:** copper metabolism, magnetic resonance angiography (mra), menkes disease, mri imaging, rare genetic disorder

## Abstract

Menkes disease is an X-linked recessive genetically inherited metabolic disease caused by an ATP7A gene abnormality that gives rise to impaired copper absorption. Copper deficiency causes symptoms such as characteristic abnormalities in the hair and vascular disorders. Brain MRI findings include a high-signal intensity in the temporal lobe white matter on T2-weighted images and delayed myelination. Intracranial arterial tortuosity seen on brain MR angiography (MRA) is one of the characteristic features of this disease. We report two cases with similar MRI findings visualized as flow voids in tortuous arteries near the central sulcus. The findings from these cases indicate that, on MRI in children, attention must be paid to intracranial arterial flow voids in patients who have not undergone MRA, particularly when Menkes disease is not suspected based on the patient's clinical course. Moreover, the findings in these cases suggest Menkes disease, indicating that they may assist in establishing the diagnosis.

## Introduction

Menkes disease (MD) is an abnormality of copper metabolism caused by an X-linked recessive genetically inherited abnormality of the ATP7A gene [1]. Although the symptoms at onset are characteristically mild during the neonatal period, a serious neurological disorder often occurs starting two to three months after birth, when maternally derived copper becomes deficient. Abnormal hair is characteristic of this disease and serves as a clue for early detection. The neurological disorder is serious and results in muscular hypotonia and intractable seizures [2]. In addition, vascular disorders caused by connective tissue abnormalities may result in subdural hematoma [3]. Brain MRI findings include marked intracranial arterial tortuosity and bending, in addition to the abnormally high signal intensity of the subcortical white matter of the temporal lobe, the former being a particularly characteristic finding in MD [4,5]. With MRI examination in children, it may be difficult to ensure that the patient remains still for a sufficient period. Consequently, it may be impossible to perform magnetic resonance angiography (MRA) during a single examination. In such cases, Menkes disease characterized by intracranial arterial tortuosity and bending may be overlooked if there is a lack of imaging findings other than abnormal vascular findings. It is therefore necessary to pay close attention to the course and shape of blood vessels on MRI images. Herein, two cases of MD with similar blood vessel courses on brain MRI that provided clues for early detection are described.

## Case presentation

Case 1

**Figure 1:**
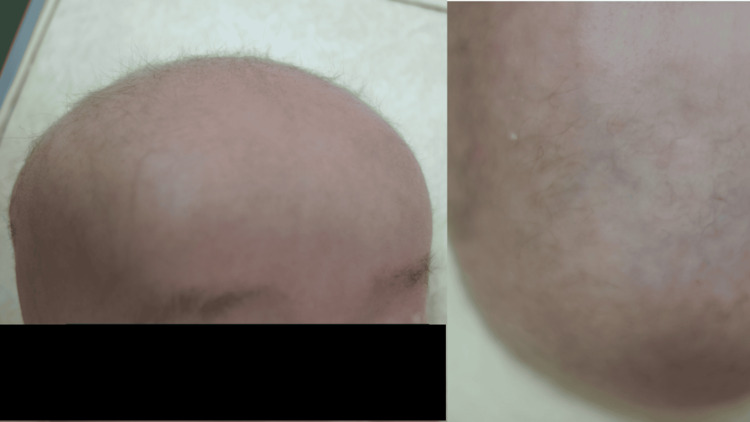
Case 1 (five-month-old male) patient’s head photographs. The hair is short, curly, and light-colored, characteristic of kinky hair.

The patient was a five-month-old male without notable abnormalities during the perinatal period. The patient was delivered by Cesarean section at 38 weeks of pregnancy due to breech presentation. His weight was 2,795 g. No neurological abnormalities were evident. Two months after birth, the patient presented at another hospital with a fever and a suckling defect and was hospitalized with diagnoses of bronchitis and urinary tract infection. At four months, the patient's neck was unable to support his head, but there were no problems with movement of the extremities. Although renal stones were seen in both kidneys on ultrasonography, hydronephrosis was not seen. Abnormal hair (kinky hair) and facial features were noted at admission to the other hospital, and the patient was therefore transferred to our hospital for detailed examination (Figure 1).

**Figure 2:**
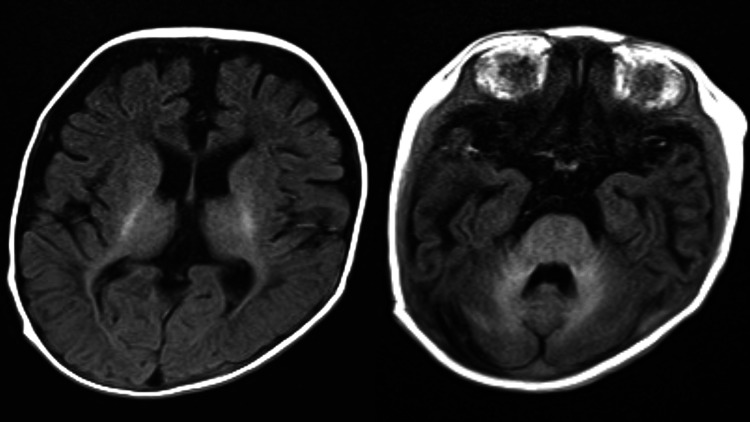
Case 1: T1-weighted images. Generalized brain atrophy is present with secondary enlargement of the ventricles and cisterns. However, there is no delayed myelination.

**Figure 3:**
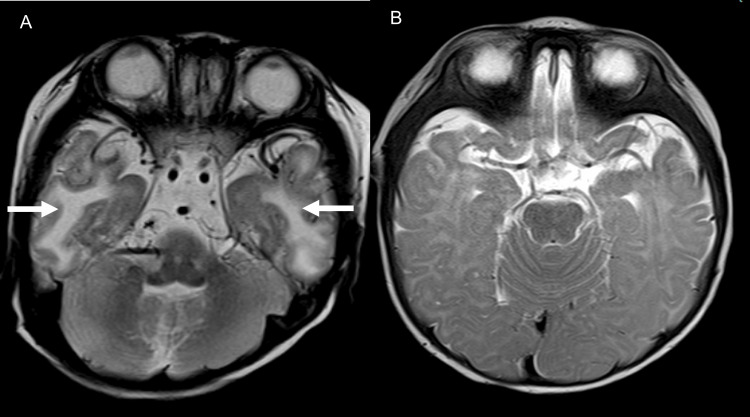
Case 1: T2-weighted image (A. T2-weighted image, B. T2-weighted image of a normal child of the same age). Compared to the normal case (B), high-signal intensity is shown in the subcortical white matter of the bilateral temporal lobes (A, arrows).

Blood tests showed low blood levels of ceruloplasmin and copper, at 10 mg/dL and 6 μg/dL, respectively. Additionally, elevated lactic acid and pyruvic acid levels were seen. Urinalysis showed a high level of beta 2-microglobulin (424 μg/L), suggesting proximal renal tubular dysfunction. Renal stones were seen in both kidneys on abdominal CT. Although brain MRI indicated that myelination was age-appropriate, generalized cerebral atrophy was seen (Figure 2). On T2-weighted images, high signal intensity was seen in the subcortical white matter of bilateral temporal lobes (Figure 3).

**Figure 4:**
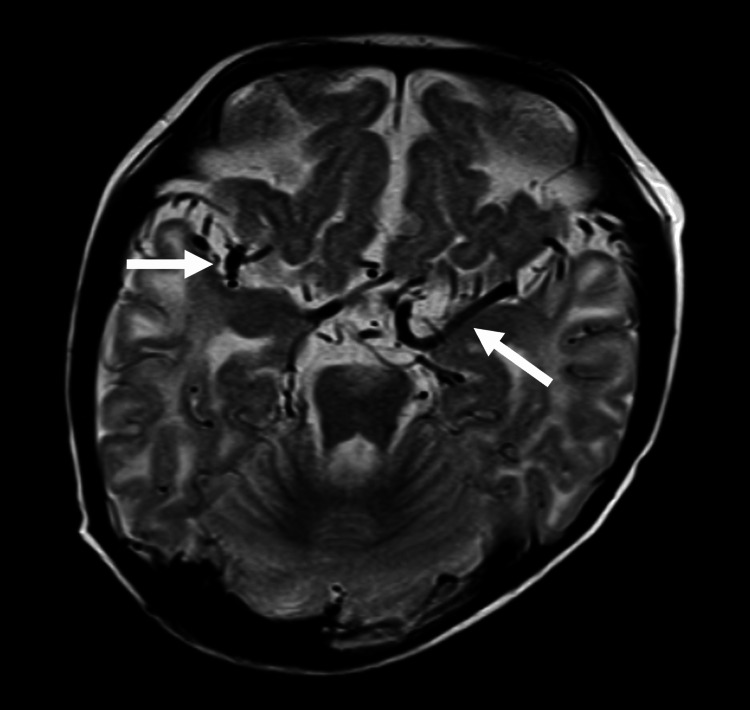
Case 1: T2-weighted image. Tortuosity and bending of the bilateral middle cerebral arteries are noted in the Sylvian fissure (arrows).

**Figure 5:**
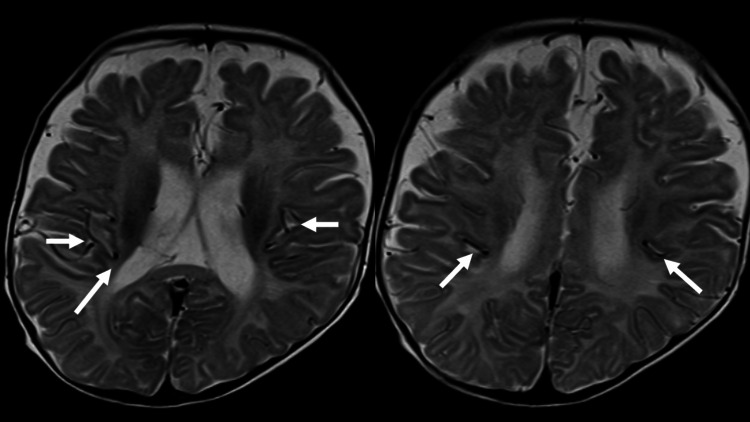
Case 1: T2-weighted images. The middle cerebral artery branches are tortuous in the sulci near the central sulcus (arrows).

In the Sylvian fissure, tortuosity and bending of the middle cerebral artery (MCA) were seen (Figure 4). In the central sulcus bilaterally and nearby sulci, flow voids of what were thought to be tortuous arterial branches were seen (Figure 5).

**Figure 6:**
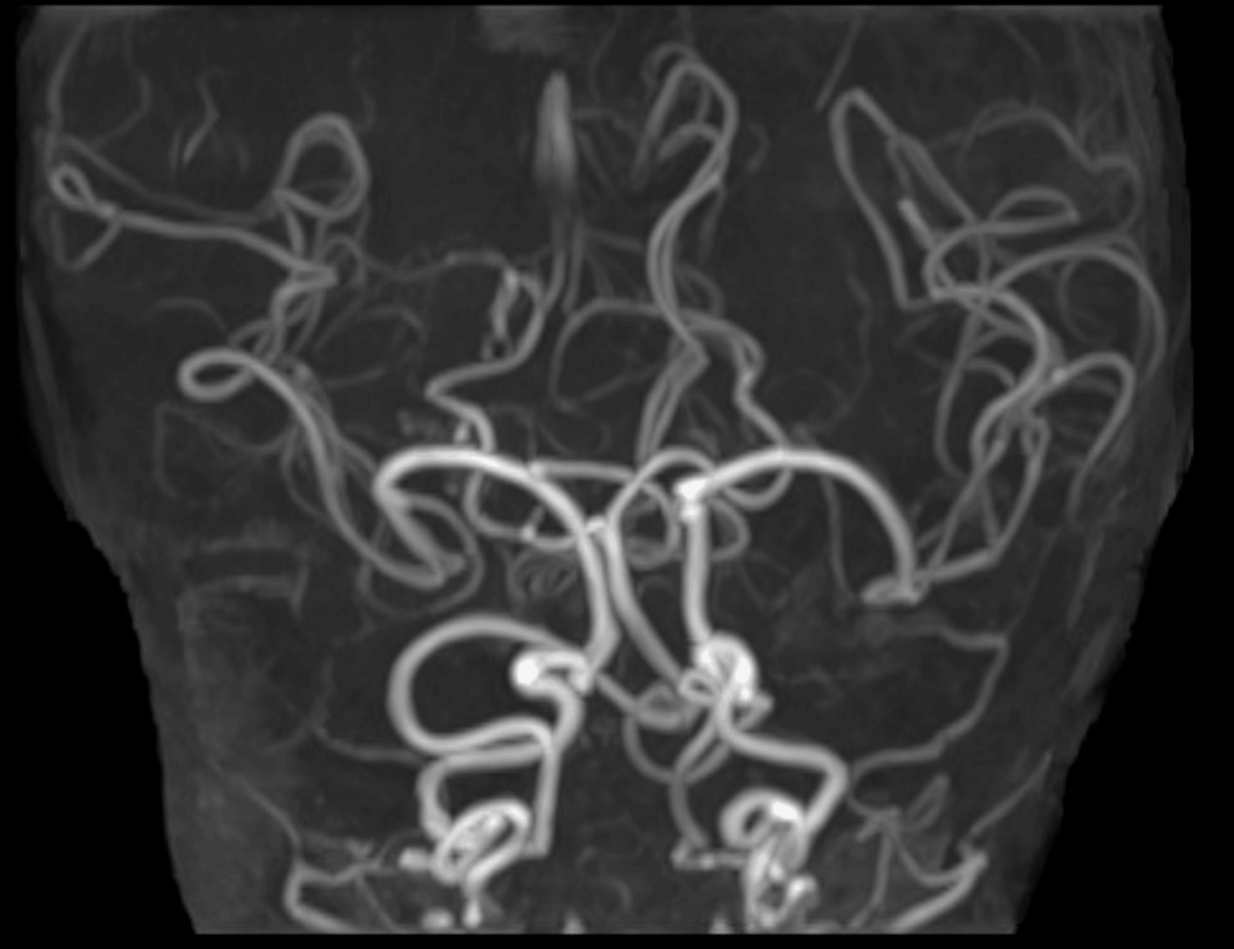
Case 1: Three-dimensional time-of-flight (TOF) MR angiogram. The tortuosity and marked angulation of the main trunks of the intracranial internal carotid, anterior cerebral, middle cerebral, and posterior cerebral arteries are revealed. Similar findings are also noted in the internal carotid artery just before entering the skull.

Time-of-flight brain MRA (maximum-intensity projection, MIP) images showed marked intracranial arterial tortuosity and bending, with pronounced tortuosity seen in peripheral branches originating from the main intracranial arteries (Figure 6). The tortuous blood vessels near the central sulcus that were identified on T2-weighted images were thought to be MCA branches.

Case 2

**Figure 7:**
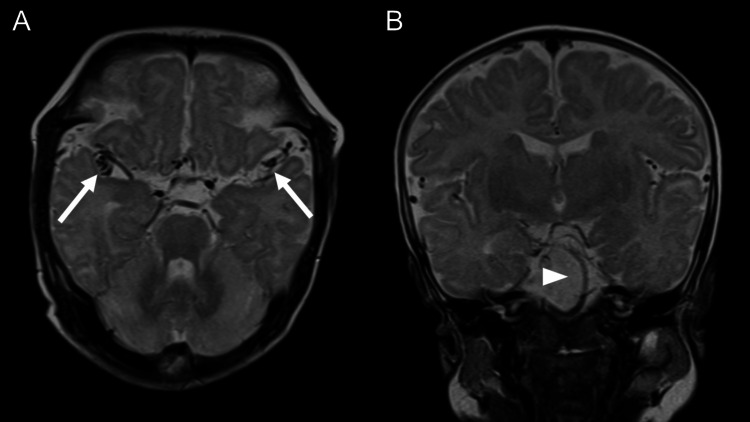
Case 2 (2-month-old male): T2-weighted images (A. Axial level of Sylvian fissure, B. Coronal image). Tortuosity of the middle cerebral artery is depicted in the Sylvian fissure (arrows). The basilar artery is also tortuous in a coronal image (arrowhead).

The patient was a two-month-old male delivered by emergency Cesarean section at 39 weeks. His weight was 3,424 g. No neurological abnormalities were evident. When the patient was two months old, findings such as poor suckling and urinary cloudiness were observed, and the patient was therefore hospitalized for treatment. Convulsive seizures were observed during hospitalization, and electroencephalography showed epileptic waves in the temporal and parietal lobes. Blood tests showed low blood levels of ceruloplasmin and copper, at 2 mg/dL and 10 μg/dL, respectively. In addition, elevated lactic acid and pyruvic acid levels were seen. Urinalysis showed a high level of beta 2-microglobulin (248 μg/L), suggesting proximal renal tubular dysfunction, as in Case 1. On brain MRI, no apparent delay in myelination was seen. Moreover, no abnormalities such as pathological atrophy or gyral dysplasia were seen. On T2-weighted images, marked intracranial arterial tortuosity and bending were seen in the Sylvian fissure and prepontine cisterns (Figure 7).

**Figure 8:**
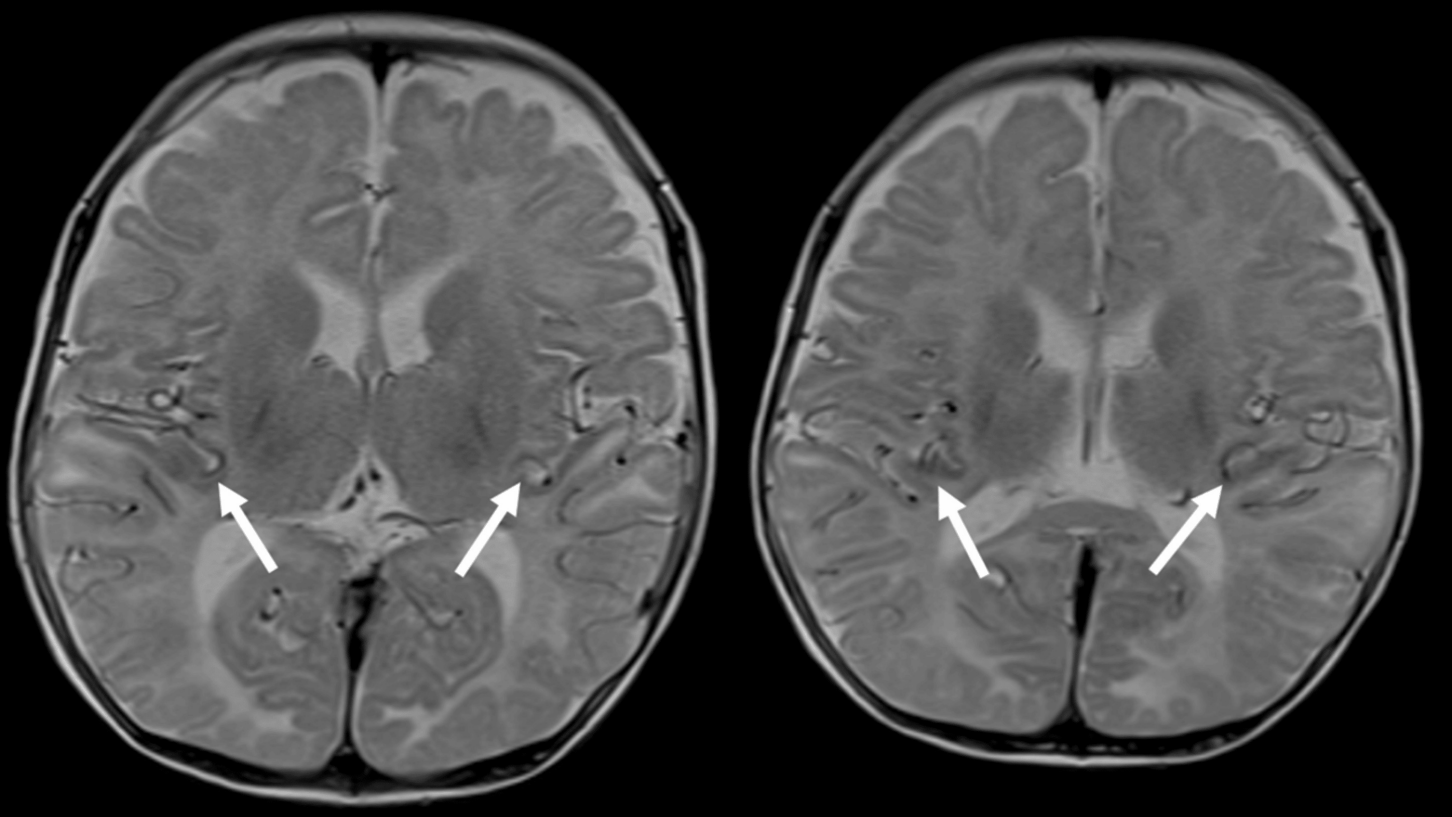
Case 2: T2-weighted images. Arterial branches show symmetrical tortuosity in the sulci near the central sulcus like that seen in Case 1 (arrows).

**Figure 9:**
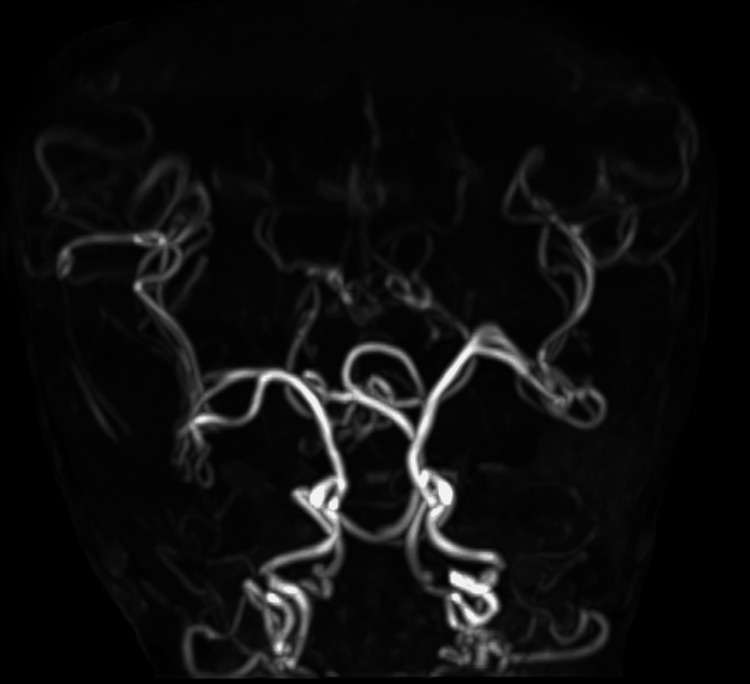
Case 2: Three-dimensional time-of-flight (TOF) MR angiogram. Like Case 1, the tortuosity and marked angulation of the main trunks of the intracranial internal carotid, middle cerebral, and posterior cerebral arteries are depicted. The internal carotid artery just before entering the skull shows similar findings.

In the central sulcus and nearby sulci, the course of roughly symmetrically tortuous cerebral artery peripheral branches was seen. This was thought to represent the dilation of MCA branches like that seen in Case 1 (Figure 8). Brain MRA (MIP) images similarly showed marked tortuosity and elongation of intracranial arteries, including the MCA (Figure 9).

## Discussion

MD was first reported in 1962 in young male patients as an X-linked, recessive, hereditary condition that characteristically manifests as abnormal hair, delayed development, and neurological disorders [6]. The cause was shown to be copper deficiency caused by impaired intestinal copper absorption. Subsequently, a copper metabolism disorder caused by dysfunction of the copper transport protein ATP7A, which results from an abnormality of the ATP7A gene on the X chromosome, was identified [4,5]. MD results in copper deficiency due to impaired copper transport caused by a copper transport protein deficit, and it leads to a variety of symptoms by reducing copper oxidase activity (e.g., cytochrome C oxidase, lysyl oxidase). Moreover, copper also accumulates at the blood-brain barrier, and copper transport to the neurons is impaired [7,8]. Decreased lysyl oxidase activity causes arterial tortuosity and dilation by impairing intimal formation in the artery wall due to abnormal collagen and elastin formation [9,10]. Arterial tortuosity is seen from early in the fetal or neonatal period and is a particularly characteristic finding in MD [11,12]. According to Gupta et al., abnormal vascular findings in MD are seen predominately in medium to large vessels [12], and cerebral infarction occurs during the disease in some cases [13]. An iliac aneurysm has also been reported as a lesion in a vessel other than an intracranial artery [14].

Known symptoms of this disease include muscular hypotonia, convulsions, and epileptic seizures. Convulsions and epileptic seizures were seen in Case 2. In our two cases, no apparent neurological abnormalities were identified at birth, and symptoms first appeared two months after birth. Neurological symptoms are not seen at birth in many patients, with symptoms such as epileptic seizures often first occurring two to three months after birth. The cases described here are consistent with this [15,16]. Characteristic abnormalities seen in blood tests are low copper and blood ceruloplasmin levels [3], both of which were seen in our cases. Elevated lactic acid and pyruvic acid levels were also seen, and these are also blood test abnormalities known to occur in MD [8]. Although no fundamental treatment has been established, subcutaneous injection of copper histidine is considered effective in improving hair abnormality and blood copper and blood ceruloplasmin levels. Although starting treatment within two months after birth is considered to prevent central nervous system disorders, starting later than two months after birth has been found not to result in improvement [3]. Connective tissue abnormalities are not thought to improve with copper histidine administration. Complications such as intractable seizures, bone fractures, and subdural hematoma often occur, bladder rupture and infection frequently occur concurrently, and the disease often becomes fatal. MD is generally diagnosed three to six months after birth, and the patient often dies before three to four years of age [3,7].

Regarding brain MRI findings in MD, cases in which myelination was delayed or in which there were no notable abnormal findings initially have also been reported [4]. In Case 2, no abnormalities of the brain parenchyma were seen, and the only abnormal findings involved the cerebral blood vessels. White matter lesions in the frontal and temporal lobes are known in the brain parenchyma in MD, with high signal intensity common on T2-weighted images and low signal intensity often seen on T1-weighted images [4,5]. As the disease stage progresses, cerebral atrophy and ventricular dilation are seen, with subdural hygroma and hematoma seen in some cases [4,5]. White matter lesions were seen in the bilateral temporal lobes in Case 1 of this case report. Cerebral atrophy was seen in Case 1, but not in Case 2.

MRA is a useful non-invasive method for evaluating vascular lesions, and it is similarly useful, particularly for testing of groups of diseases that result in vascular abnormalities during the infant/neonatal period. An advantage of MRI is that, unlike CT, there is no radiation exposure or contrast-associated risk. Non-contrast-enhanced MRA is also useful in groups of diseases, such as MD, that manifest as cerebrovascular abnormalities resulting from genetic abnormalities. However, MRI, including MRA, generally has the disadvantage of long acquisition times. Because of their high spatial resolution, MRA examinations using the time-of-flight method are commonly used for non-contrast-enhanced MRA in the head and neck region. However, the imaging time is long, around five minutes, making this method susceptible to artifacts resulting from a child's movements. On routine examinations, MRI of the brain parenchyma (e.g., FLAIR image and diffusion-weighted imaging) is performed first, and this is the case for MRI examinations in children, as well as adults. MRA is therefore often performed subsequently. Particularly with pediatric patients, maintaining stillness for a long time is difficult, and MRA images cannot be obtained due to the patient's movement. Although sedation can be performed before examination, it places a burden on a pediatric patient, and its usefulness must therefore be adequately weighed. In diseases that show cerebral arterial abnormalities, another option is to perform imaging with priority given to MRA. If these diseases are assumed not to be present before the examination, MRA is not performed as a screening test in some cases. Therefore, when MRA has not yet been performed, imaging findings that can suggest abnormalities in blood vessels, particularly their caliber and course, shown as flow voids on T2-weighted images, may be clues for planning the imaging procedure.

In the two present cases with suspected MD, similar symmetrical tortuosity of intracranial arteries that traveled along the central sulcus bilaterally and nearby cerebral sulci were seen as flow voids on T2-weighted images. Comparison with the original MRA image indicated tortuosity of arteries running in or adjacent to the central sulcus, which are branches of the MCA. Symmetrical tortuosity was not seen in intracranial arterial branches at other sites; it was characteristically observed in branches in the territory of the MCA. The course of a blood vessel is often distinctly visualized as a flow void on T2-weighted images, and tortuosity of the proximal MCA can be readily observed mainly in the Sylvian fissure. Regarding brain MRI findings in MD, there have been numerous reports that have described abnormalities of the brain parenchyma and abnormalities of the course of arteries seen on MRA [17,18]. Although limited to just two cases, similar arterial tortuosity was observed in the same location on T2-weighted images, not on MRA, representing an intriguing MRI finding. We believe that this may warrant further attention as a potential MRI feature of MD other than MRA. These findings on T2-weighted images appear to be suggestive of MD and to be a useful clue for diagnosis. Although diseases that result in intracranial arterial tortuosity and are caused by genetic abnormalities, including Marfan's syndrome and vascular Ehlers-Danlos syndrome, there have been no reported cases of the symmetrical tortuosity of MCA branches seen in the present cases. Although these are only two cases, the findings obtained by MRI provided clues for differentiating MD from these other disease groups.

As mentioned, copper histidine administration is considered an effective treatment for this disease, and starting treatment within two months after birth can prevent neurological disorders [3]. Early diagnosis of MD is therefore needed. However, early diagnosis is considered difficult because no abnormalities are seen immediately after birth in some cases, and there are individual differences in the physical manifestations of MD, such as kinky hair [18]. In addition to blood tests and genetic testing, imaging examinations are also important for the early diagnosis of MD. In routine MRI examinations for early diagnosis, the presence or absence of abnormalities of the intracranial arteries is considered important. The cases described suggest that focusing on the course of the intracranial arteries in the central sulcus and its vicinity may provide clues for the early diagnosis of MD.

## Conclusions

In the two cases of Menkes disease we reported, similar vascular tortuosity was noted on T2-weighted images. This finding was considered characteristically suggestive of Menkes disease on MRI images other than MRA. Early diagnosis of MD is difficult due to individual differences in physical symptoms in some cases. The results of the present cases suggest that findings suggestive of MD obtained on MRI acquired before MRA could be useful for the early diagnosis of MD.
